# A New Screening Tool (BAMSA) for Sleep Apnea in Male Professional Truck Drivers

**DOI:** 10.3390/jcm13020522

**Published:** 2024-01-17

**Authors:** Riikka Huhta, Mariusz Sieminski, Kari Hirvonen, Eemil Partinen, Markku Partinen

**Affiliations:** 1 Department of Clinical Neurosciences, University of Helsinki, Clinicum, 00140 Helsinki, Finland; eemil.partinen@gmail.com (E.P.); markku.partinen@helsinki.fi (M.P.); 2 Helsinki Sleep Clinic, Terveystalo Healthcare, 00380 Helsinki, Finland; 3 Department of Emergency Medicine, University of Gdansk, 80-214 Gdansk, Poland; mariusz.sieminski@gumed.edu.pl; 4 Neurotest Tampere Oy, 33101 Tampere, Finland; kari.hirvonen@neurotest.fi

**Keywords:** sleep apnea, professional drivers, snoring, screening, sleep disordered breathing

## Abstract

Obstructive sleep apnea (OSA) is common in professional truck drivers. It is important that OSA is recognized since undiagnosed and/or untreated sleep apnea is a risk factor for sleepiness-related traffic accidents. In this study, we developed a new simple tool to screen for obstructive sleep apnea (OSA) in this population. Altogether, 2066 professional truck drivers received a structured questionnaire. A total of 175 drivers had a clinical examination and were invited to participate in sleep laboratory studies, including cardiorespiratory polygraphy. We studied associations of different risk factors with the presence of sleep apnea. We established a new simple screening tool for obstructive sleep apnea (OSA) that was compared to other existing screening tools. A total of 1095 drivers completed the questionnaire. Successful cardiorespiratory polygraphy was obtained for 172 drivers. Full data were available for 160 male drivers included in the analyses. The following five risk factors for sleep apnea formed the BAMSA score (0 to 5): BMI > 30 kgm^−2^, age > 50 years, male gender, snoring at least one night per week, and the presence of apnea at least sometimes. BAMSA showed a sensitivity of 85.7% and a specificity of 78.8% in detecting AHI ≥ 15 when using a cut-off point of 4, and the ROC area was 0.823. BAMSA is a sensitive and easy-to-use tool in predicting obstructive sleep apnea in male professional drivers.

## 1. Introduction

Obstructive sleep apnea (OSA) is a common disorder causing daytime sleepiness. In the general population, the prevalence of sleep apnea defined by AHI ≥ 5 ranges from 9 to 38% in men and 1 to 17% in women [1,2]. It is even more prevalent among professional truck drivers. In recent studies, prevalence figures as high as 28–78% have been reported [3,4,5,6,7]. Daytime sleepiness increases the risk of traffic accidents. In Finland, driver sleepiness/fatigue is a contributing factor in 17–19% of fatal vehicle accidents [8,9]. In 2013, the European Driving License Committees Working Group estimated that sleep apnea and narcolepsy together cause a relative risk elevation of 271% (RR = 3.71) for traffic accidents, and this is mostly due to sleep apnea [10].

OSA is classified as mild, moderate, or severe according to the apnea–hypopnea index (AHI). European Union directives rule that daytime sleepiness should be assessed annually in professional drivers with at least moderate sleep apnea (AHI ≥ 15). Obstructive sleep apnea syndrome is a treatable condition, e.g., by continuous positive airway pressure (CPAP) [11,12]. Hence, it is important to detect the possibility of OSA via occupational health care. Furthermore, evidence shows that CPAP treatment reduces the risk of motor vehicle accidents [13,14].

Previously, different tools for predicting sleep apnea have been described. Some of these require oximetry data [15,16,17] and some require clinical evaluations of anatomy (for example the Mallampati score) [18], such as the DES-OSA score [19]. In addition, the automatic detection of OSA via speech signals has been studied [20]. Different prediction tools using questionnaires and certain measurements, for example, neck circumference or systolic blood pressure, have emerged. However, these tools have not included clinical evaluation. Among these are the SAS score [21], No-Apnea [22], the STOP questionnaire [23], STOP-BANG [24,25], the NoSAS score [26,27], and the Four-Variable Screening Tool (Four-V) [28].

Of the available prediction tools, the Berlin questionnaire [29,30,31], a clinical prediction rule presented by Miranda Serrano et al. [32], and the Multivariable Apnea Prediction (MAP) index [33] are more easily accomplished since they only include questions to be answered by the patient and the only measurement required is the patient’s body mass index (BMI).

Also, the Epworth Sleepiness Scale (ESS) has been suggested to predict the probability of sleep apnea, and several studies with different outcomes have been published previously [34,35]. However, the ESS did not appear to correlate with sleep disordered breathing (SDB) in women, and the prediction value in men was modest at best, with an average ESS score of 9.1 for no SDB and 10.1 for severe SDB [36]. Several studies have compared the accuracy of different predictive tools, and meta-analyses have also been carried out [37,38,39]. Quaranta et al. also established a tool called the Driver Sleepiness Score (DSS) for the evaluation of sleepiness in drivers with suspected OSA [40].

There is still a need for an easy and cheap method to recognize individuals who should undergo sleep studies, especially in occupational health care. In this study, we wanted to estimate the usefulness of various screening tools in detecting OSA and to establish a new simple tool that does not require any clinical examination by a clinician for the screening of OSA.

## 2. Materials and Methods

### 2.1. Methods

A structured questionnaire was sent to 2066 professional truck drivers living in Southern Finland, as described previously [7]. We selected the subjects from the Finnish Truck Drivers’ (Rahtarit ry) registry. A total of 175 of these drivers had a clinical examination. The sleep studies included cardiorespiratory polygraphy, daytime sleepiness tests, and a maintenance of wakefulness test (MWT). Sleep studies were carried out successfully for 172 drivers. Of these drivers, we only included those who had complete information in this BAMSA study so that we could also compute their STOP-BANG, NoSAS, ESS, and the Berlin scores. In total, 160 men were included.

### 2.2. Questionnaire

Demographic variables, including age, weight, height, and neck circumference, were obtained via the questionnaire and again during the medical visit. BMI values were highly correlated (r = 0.9675). The BMIs of participants were somewhat higher during the medical visits than the BMIs provided in the questionnaires (BMI (visit) = 2.36 + 0.913 × BMI (questionnaire); R^2^ = 0.9386). The correlation of neck circumference (NC) between the visit and questionnaire was less significant (r = 0.733; NC (visit) = 18.47 + 0.548 × NC (questionnaire); R^2^ = 0.537). We used the measured BMI and neck circumference data since they were always available. Age was taken as the age at the time of the visit for this study.

All subjects completed the questionnaire, which was modified from the Basic Nordic Sleep Questionnaire (BNSQ) [41]. In addition, they answered questions regarding their driving history, sleepiness while driving, and other driving-related items.

The question concerning snoring (from the BNSQ) was: “Do you snore while sleeping (ask other people if you are not sure)?”. The history of subjective sleep apnea (from the BNSQ) question was phrased as: “Have you had breathing pauses (sleep apnea) at sleep (have other people noticed that you have pauses in respiration when you sleep)?”.

The question regarding quality of snoring was: “How do you snore (ask other people about the quality of your snoring)?” Response options were: (1) “I do not snore”, (2) “my snoring sounds regular and it is of low voice”, (3) “it sounds regular but rather loud”, (4) “it sounds regular but it is very loud (other people hear my snoring in the next room)”, and (5) “I snore very loudly and intermittently (there are silent breathing pauses when snoring is not heard and at times very loud snorts with gasping”.

The question concerning witnessed apneas was: “Have you had breathing pauses (sleep apnea) at sleep (have other people noticed that you have pauses in respiration when you sleep)?” Response alternatives were: (1) “never or less than once per month”, (2) “less than once per week”, (3) “on 1–2 nights per week”, (4) “on 3–5 nights per week”, and (5) “every night or almost every night”.

We also asked about daytime fatigue, tiredness, and sleepiness. In addition, the Epworth Sleepiness Scale questionnaire was completed by the respondents.

### 2.3. The Berlin Score, STOP-BANG, NoSAS, and ESS Criteria

The Berlin Questionnaire includes questions from three categories [29]. The first includes questions related to snoring and breathing pauses, the second includes tiredness and falling asleep while driving, and the third concerns high blood pressure and body mass index (BMI). The subject is categorized as being in a high-risk group when at least two positive categories are identified (this means answering positively to two questions from categories one and two, one question in category three, or having a BMI > 30).

The 8-item STOP-BANG score was computed [24]. It includes questions of snoring, tiredness, breathing pauses, high blood pressure, BMI, age, sex, and neck circumference, with options of yes or no. The risk for sleep apnea increases with higher scores. We computed the results with the older STOP-BANG criteria and also the new criteria, which has different neck circumference criteria for males and females [42].

The NoSAS score is a screening tool that has been used previously [26]. It ranges from 0 to 17, and includes questions on neck circumference, BMI, snoring, sex, and age; with 8 points or more, the individual is at high risk for OSA.

The Epworth Sleepiness Scale (ESS) has also been presented as a possible predictor of OSA [34]. In the ESS questionnaire, the subject is asked to evaluate the probability of falling asleep in eight different situations and the total score ranges from 0 to 24.

### 2.4. Design of the BAMSA Criteria

First, we computed sensitivity, specificity, and the receiver operating characteristic (ROC) for detecting obstructive sleep apnea using different screening tools. We also used our empirical experience in diagnosing patients with sleep apnea since the late 1970s. We wanted to design a tool that is as simple as possible yet sensitive and, in particular, sufficiently specific. We also wanted to have a screening tool based mainly on anamnestic information using semi-quantitative questions instead of dichotomic yes/no alternatives for the occurrence of snoring and of sleep apnea [41]. The reason for this is that almost all humans snore at least sometimes if they are sleeping in the supine position and if they have been drinking alcohol. For this reason, “snoring—yes” differs from “snoring at least one night per week”. For sleep apnea the cut-off point for “yes” was: having witnessed breathing pauses (apneas) at least one night per month.

The new BAMSA screening tool consists of 5 questionnaire (interview) items: (1) BMI > 30 kgm^−2^ (no = 0, yes = 1); (2) Age > 50 years (no = 0, yes = 1); (3) gender (female = 0, Male = 1); (4) Snoring at least one night per week (from the BNSQ; no = 0, yes = 1); and (5) having had breathing pauses at least sometimes (Apneas) during sleep (BNSQ response > 1; no = 0, yes = 1). Please see the Appendix A for the BAMSA questionnaire.

### 2.5. Clinical Examination

All subjects who were monitored in the sleep laboratory underwent a thorough medical examination. Weight and height were measured. The shape of the hard and soft palate was tabulated as well as the size and shape of the tonsils, tongue, jaw (micrognathia, retrognathia), and bite (overjet, signs of bruxism, etc.). The clinician measured the neck and waist circumferences and tabulated the cricomental space using three classifications: normal, slightly abnormal, and abnormal. Pulse rate and blood pressure were also measured and a neurological examination was performed.

### 2.6. Sleep Recordings

All 160 subjects underwent full-night cardiorespiratory polygraphic sleep recordings, as described earlier [7]. We recorded nasal airflow, and thoracic and abdominal respiratory movements, as well as snoring sounds, sleep position, pulse, and oxygen saturation. We used a limit of ≥4% from the pre-event baseline for oxygen desaturations as defined by the AASM 1999 Type A definition/AASM 2.2 Criteria 1B for the detection of hypopnea [43,44]. The 1999 rules with the 4% desaturation criteria have been reported to provide essentially similar results to the current AASM rules [44]. We categorized the severity of obstructive sleep apnea (OSA) into four groups in terms of the apnea–hypopnea index (AHI): AHI < 5/h (normal/no sleep apnea), 5 ≤ AHI < 15 (mild), 15 ≤ AHI < 30 (moderate), and AHI ≥ 30 (severe).

MWT was performed following the same method as has previously been described with four 40 min sessions [45]. The session was discontinued if the subject fell asleep (after three consecutive epochs (30 s) of stage 1 sleep or after one epoch of any other sleep stage).

### 2.7. Statistics

We carried out statistical analyses using STATA versions 15.1 and 17.0 (StataCorp, College Station, TX, USA). We used medians, means, standard deviations (SD), and ranges to describe distributions. We tested statistical differences between groups using the Student’s t-test, Mann–Whitney U test, or the Kruskal–Wallis test, depending on the number of groups and nature of the distribution. We tested the normality of the distribution using the Shapiro–Wilk test [46]. Pearson’s chi-square test was computed from the cross-tabulations. Receiver operating characteristic (ROC) analysis and the area under the curve were computed when applicable. Sensitivity, specificity, positive predictive values (PPVs), negative predictive values (NPVs), the positive likelihood ratio (LR+), and the negative likelihood ratio (LR−) were computed using the diagt command of STATA [47]. Odds ratios (ORs) of a test with a specific cut-off point (LR+/LR−) were computed. Before starting the project, we carried out power analyses (STATA and nQuery) using different assumptions and different AHI cut-off points. We used a significance level of 0.05 for the error probabilities and 90% power. As AHI ≥ 5 is commonly found in men and especially in obese men, we would have needed more than 300 drivers with OSA to achieve 90% power. Due to financial constraints, polysomnograms were not carried out for all participants. Consequently, we also computed the power for other end-points and ended up with 160 subjects. Using a cut-off point of AHI 15, only 12 subjects would have been required in the OSA group and also in the control group in order to achieve 90% power with an alpha of 0.05.

### 2.8. Ethical Considerations

The ethical committee of the Helsinki University Hospital approved this study. This study obeyed the guidelines and instructions of the Declaration of Helsinki. Every subject provided written informed consent. All information was strictly confidential and access to all data was restricted only to investigators in this study. If moderate or severe sleep apnea was diagnosed, the subjects had the opportunity to give permission for their data to be sent to the public healthcare system in order for them to receive treatment for sleep apnea. The employers of the truck drivers did not have access to any of the data.

## 3. Results

Characteristics of the drivers

Altogether, 160 subjects were men. Baseline characteristics (age, BMI, waist circumference, and neck circumference) are introduced in Table 1. A total of 39 men (24.4%) were over 50 years old; 49 men (30.6%) were obese with a BMI > 30 kgm^−2^; 31 men (19.4%) had a neck circumference > 43 cm. For 74 men (46.25%), their waist circumference was >100 cm, and for 36 men (23.1%), their waist circumference was >110 cm. A total of 27 men (16.9%) used antihypertensive medication and/or had a diagnosis of arterial hypertension.

Snoring at least 3 nights per week was reported by 83 men (51.9%) and this also includes habitual snoring occurring almost every night, which was reported by 70 men (43.75%). A total of 27 men (16.9%) reported that they never snore. Loudness (quality) of snoring was associated with frequency of snoring history. All men reporting very loud and intermittent snoring (n = 69) occurring at least 3 nights per week. Sixty of these men (87%) were habitual snorers.

Breathing pauses (apneas) occurring at least sometimes were reported by 86 men (53.75%). Apneas occurring at least 3 nights per week were reported by 53 men (33.1%) and this also included those 36 men (22.5%) who reported having apneas almost every night. A total of 34 (94.4%) of these 36 men reported snoring almost every night, and 74 men (46.3%) reported that they have never had witnessed apneas.

The apnea–hypopnea index (AHI) was ≥5 in 70 drivers (43.75%), ≥15 in 17.5% (n = 28), and ≥30 in 7.5% (n = 12) of these 160 subjects. A total of 53.9% of those snoring at least three nights per week had an AHI ≥ 5. Only one of the drivers without any prior history of snoring had an AHI ≥ 30. His BMI was 32.7 kg/m^2^ and he was 57 years old. On the other hand, 33.3% of these drivers had an AHI ≥ 5, and 7.4% (n = 2) had an AHI ≥ 15 (one had an AHI = 52 and the other had an AHI = 18). In other words, the simple absence of snoring did not completely exclude severe sleep apnea.

Altogether, 33 men (20.6%) reported tiredness occurring at least three days per week. The mean ESS score was 8.7 (SD 3.8, median 9, range 1–19). The ESS score was >10 in 47 (29.4%) and >15 in 8 (5.0%) drivers. A total of 28.6% of those who had an AHI ≥ 15 had an ESS score > 10, and 29.6% of those who had an AHI < 15 had an ESS score > 10. An ESS score of over 10 points was observed for 31.4% of drivers who had an AHI ≥ 5 and for 27.8% of those who had an AHI < 5. The ESS score was, on average, 8.6 (SD 3.7, median 8) among those who had an AHI ≥ 5 and 8.7 (SD 3.8, median 8.5) among those who had an AHI < 15.

Altogether, 156 of the participants performed the Maintenance of Wakefulness Test (MWT) successfully. A total of 20.5% of these participants had an MWT score < 19.4 min, which is considered abnormal [45]. In addition, 12.2% of the participants had both an MWT score < 19.4 min and an AHI score ≥ 5. A total of 7.7% had both an MWT score < 19.4 min and an AHI score ≥ 15.

Performance of different screening tools

The ROC area for the Berlin score was 0.660 for the presence of sleep apnea (AHI ≥ 5). The ROC area of the original STOP-BANG score was 0.724. The ROC area of the STOP-BANG score with the new neck circumference criteria (STOP-BANGnew) was 0.734. The ROC area for the NoSAS score was 0.698. The ROC area for the ESS score was 0.524 (for ESS score ≥ 11, the ROC area was 0.518). The ROC area of BAMSA for AHI ≥ 5 was 0.741. BAMSA was most effective at detecting AHI ≥ 5 (see Table 2). The ROC area of the Epworth Sleepiness Scale was close to 0.5.

For detecting an AHI score ≥ 15, the sensitivity and specificity of BAMSA with a cutoff point of 3 were 100% and 47%, respectively (see Table 3). With a BAMSA cut-off point of 4, the sensitivity and specificity were 85.7% and 78.8%, respectively (see Table 2).

**Figure 1:**
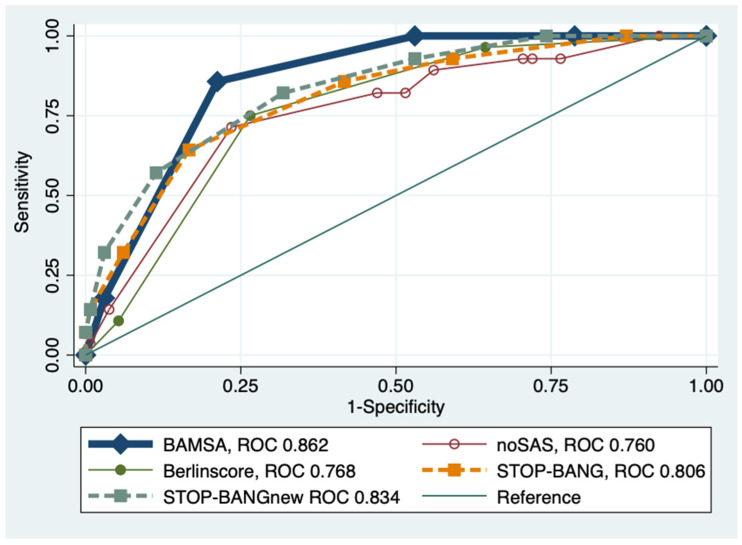
Comparison of the ROC values of different screening tools (AHI ≥ 15). AHI: Apnea–hypopnea; ROC: Area under the receiver operating curve.

Using AHI ≥ 15, the overall ROC area for BAMSA was 0.862. The ROC area of the BAMSA score was more favorable in terms of statistical significance, better than that of the original STOP-BANG (*p* = 0.085), the Berlin score (*p* = 0.0005), ESS (*p* < 0.0001), and NoSAS (*p* = 0.027). There was no statistically significant difference between BAMSA and the modified STOP-BANGnew score with the new neck circumference criteria (*p* = 0.342) (see Figure 1).

As all subjects were men, we analyzed the data by excluding gender from the formulas. The ROC areas of the different screening tools did not change. We wanted to keep male gender in the BAMSA questionnaire since it is a known risk factor for sleep apnea and is common among professional truck drivers.

We also calculated the performance of BAMSA when including the MWT data. Using AHI ≥ 5 and MWT < 19.4 min, the ROC area for BAMSA was 0.693 when using the cut-off point of 3. In this case, the sensitivity was 94.7% and the specificity was 43.8%. Using these same criteria and a BAMSA cut-off point of 4, the ROC area was 0.677. In this case, the sensitivity was 63.2% and the specificity was 72.3%.

## 4. Discussion

Our aim was to create and validate a screening tool for professional drivers that is easy to use without any contact with the healthcare system. We introduce BAMSA (0 to 5 points), a simple five-question tool that is shown to be at least as sensitive and more specific than the other tested screening tools in detecting obstructive sleep apnea. It detects mild sleep apnea, especially moderate to severe sleep apnea, very effectively. It includes four questions (no = 0 points, yes = 1 point) and the measurement of BMI (>30 kgm^−2^ = 1 point) and is easy to use, allowing for self-measurement at home by subjects themselves. Using this prediction tool, it is also easy for a clinician to determine which patients to send for sleep laboratory studies. The ROC area of BAMSA for AHI ≥ 5 was 0.741. BAMSA was even more effective at detecting moderate and severe sleep apnea, and the ROC area of BAMSA for AHI ≥ 15 was 0.862. When using the cut-off point of 4 points, the sensitivity of BAMSA in detecting AHI ≥ 15 was 85.7% and the specificity was 78.8%.

STOP-BANG is one of the best validated and most commonly used screening tools [24]. Recently the criteria for STOP-BANG, particularly concerning the neck circumference criteria, have been updated [42]. It includes measurement of neck circumference, and the criteria differ with regard to gender. The scoring of the original [24] and also the new STOP-BANG [42] are somewhat more complicated than the scoring of BAMSA, and it includes different options for evaluating a subject in a high-risk group. The NoSAS score [26] includes sex, age, BMI, snoring, and neck circumference, and it also has a rather complicated scoring system. The Berlin Questionnaire is another previously established screening tool [29] that does not require any measurements, but again, the scoring is complicated. The Epworth Sleepiness Scale is a widely used questionnaire to test for subjective daytime sleepiness. It has been suggested as an option for predicting the risk of sleep apnea [34], but the results have not been promising [36]. In fact, Lee et al. suggested that the ESS should not be used in clinical settings [48]. Also, in our study, the ESS performed poorly and its predictive value for sleep apnea was close to tossing a coin.

A screening tool should be sensitive with a good negative predictive value to assure that there is a low incidence of false negatives. At the same time, it should be specific enough to avoid a high rate of false-positive suspicion of sleep apnea—to avoid unnecessary polygraphic sleep recordings, and to avoid causing unnecessary anxiety for the screened subjects. In this study, BAMSA showed a good sensitivity in detecting clinically relevant OSA (AHI ≥ 15) at 86.2% (cut-off point 4) or even 100% (cut-off point 3). Specificity was also better than that of the other tested prediction tools (Table 1 and Table 2). This means that BAMSA not only detects almost all cases, but it also detects them with a higher degree of accurately than the other prediction tools. BAMSA also performed well when the MWT results were considered. However, as the studies of the other prediction tools had not used this method, we did not use these values for comparisons.

The strengths of our study include the fact that our population of professional truck drivers consisted of individuals who were assumed to be healthy as well as those with suspected sleep apnea. In addition, our study population was rather large. The evaluation of the occurrence of snoring is based on a quantitative time scale instead of purely subjective evaluation [41]. The words such as “sometimes” and “often” may mean different things for different people, while for example, “on three nights per week” means exactly the same for everybody. Many of the populations evaluated in previous studies included only those who had sleep studies as a result of suspected sleep apnea or another sleep disorder [19,27,49,50,51]. Also, as far as we know, this is the first screening tool to be used to screen professional drivers.

There are also limitations of our study. Our study population consisted of male truck drivers only. Therefore, our results cannot be generalized to female populations nor populations other than professional drivers. Consequently, we have also started to validate BAMSA in other populations, including women. However, we can already conclude that BAMSA can be used as a screening tool for clinically significant obstructive sleep apnea in male populations. We used respiratory polygraphy instead of full polysomnography, which might have found additional subjects with mild sleep apnea. However, we are confident that all subjects with clinically relevant OSA were identified.

BAMSA is a valid tool that may be used instead of, or in combination with other screening tools, for example STOP-BANG, in making inferences on prior probability of sleep apnea. Although BAMSA is working well in male populations, we need to study larger populations including women and younger subjects before we can conclude that it works well in all clinical settings.

## 5. Conclusions

In conclusion, we have created a sensitive and specific screening tool for obstructive sleep apnea in male professional drivers. BAMSA is easy to use and simpler compared with the other established screening tools. BAMSA only consists of five yes or no questions. The cut-off point of three seems to be able to identify all cases of OSA and, if using a cut-off point of four, the specificity is high. Using BAMSA can help to identify professional male truck drivers who are at risk of OSA, especially those who should be referred for sleep laboratory tests.

## Figures and Tables

**Table 1:** Baseline characteristics of the subjects.

	Mean	SD	Median	Range
Age (years)	42.0	9.3	41.4	20–58
BMI (kgm^−2^)	28.3	5.1	27.3	19.4–46.2
Waist circumference (cm)	41.2	3.1	41	35–52
Neck circumference (cm)	101.4	15.2	99.5	67.5–160

**Table 2:** Calculated values for screening tools in detecting AHI ≥ 5.

	SENS	SPEC	PPV	NPV	LR+/LR−	OR	ROC
BAMSA ≥ 5	11.4%	98.9%	88.9%	58.9%	10.3/0.896	11.5	0.552
**BAMSA ≥ 4**	**57.1%**	**86.7%**	**76.9%**	**72.2%**	**4.29/0.495**	**8.67**	**0.719**
***BAMSA ≥ 3***	***78.6%***	***52.2%***	***56.1%***	***75.8%***	***1.64/0.41***	***4.01***	***0.654***
STOP-BANG ≥ 4	65.7%	63.3%	58.2%	70.4%	1.79/0.541	3.31	0.645
STOP-BANG ≥ 3	80%	46.7%	53.8%	75%	1.5/0.429	3.5	0.633
STOP-BANGnew ≥ 4	60%	74.4%	64.6%	70.5%	2.35/0.537	4.37	0.672
*STOP-BANGnew ≥ 3*	*77.1%*	*53.3%*	*56.3%*	*75%*	*1.65/0.429*	*3.86*	*0.652*
Berlin high risk	58.6%	73.3%	63.1%	69.5%	2.2/0.565	3.89	0.660
NoSAS ≥ 7	85.7%	34.4%	50.4%	75.6%	1.31/0.415	3.15	0.601
NoSAS ≥ 8	75.7%	48.9%	53.5%	72.1%	1.48/0.497	2.98	0.623
NoSAS ≥ 9	70%	53.3%	53.8%	69.6%	1.5/0.563	2.67	0.617
ESS ≥ 16	8.57%	97.8%	75%	57.9%	3.86/0.935	4.13	0.532
ESS ≥ 11	31.4%	72.2%	46.8%	57.5%	1.13/0.949	1.19	0.518
Tiredness at least one day per week	87.1%	20%	45.9%	66.7%	1.09/0.643	1.69	0.536

SENS: Sensitivity; SPEC: Specificity; PPV: Positive predictive value; NPV: Negative predictive value; LR: Likelihood ratio; OR: Odds ratio (LR+/LR−); ROC: Area under the receiver operating curve.

**Table 3:** Calculated values for screening tools in detecting AHI ≥ 15.

	SENS	SPEC	PPV	NPV	LR+/LR−	OR	ROC
BAMSA ≥ 5	17.9%	97%	55.6%	84.8%	5.89/0.847	6.96	0.574
**BAMSA ≥ 4**	**85.7%**	**78.8%**	**46.2%**	**96.3%**	**4.04/0.181**	**22.3**	**0.823**
**BAMSA ≥ 3**	**100%**	**47%**	**28.6%**	**100%**	**1.89/0**		**0.735**
STOP-BANG ≥ 4	85.7%	58.3%	30.4%	95.1%	2.06/0.245	8.4	0.720
STOP-BANG ≥ 3	92.9%	40.9%	25%	96.4%	1.57/0.175	9	0.669
STOP-BANGnew ≥ 4	82.1%	68.2%	35.4%	94.7%	2.58/0.262	9.86	0.752
*STOP-BANGnew ≥ 3*	*92.9%*	*47%*	*27.1%*	*96.9%*	*1.75/0.152*	*11.5*	*0.699*
Berlin high risk	78.6%	67.4%	33.8%	93.7%	2.41/0.318	7.59	0.730
NoSAS ≥ 7	92.9%	29.5%	21.8%	95.1%	1.32/0.242	5.45	0.612
NoSAS ≥ 8	89.3%	43.9%	25.3%	95.1%	1.59/0.244	6.53	0.666
NoSAS ≥ 9	82.1%	48.5%	25.3%	92.8%	1.59/0.368	4.33	0.653
ESS ≥ 16	7.14%	95.5%	25%	82.9%	1.57/0.973	1.62	0.513
ESS ≥ 11	28.6%	70.5%	17%	82.3%	0.967/1.01	0.954	0.495
Tiredness at least one day per week	89.3%	18.2%	18.8%	88.9%	1.09/0.589	1.85	0.537

SENS: Sensitivity; SPEC: Specificity; PPV: Positive predictive value; NPV: Negative predictive value; LR: Likelihood ratio; OR: Odds ratio (LR+/LR−); ROC: Area under the receiver operating curve.

## Data Availability

Research data are available on request from the authors without personal data for study purposes only.

## Abbreviations

AHI	Apnea Hypopnea Index
BNSQ	Basic Nordic Sleep Questionnaire
CPAP	Continuous Positive Airway Pressure
ESS	Epworth Sleepiness Scale
LR	Likelihood Ratio
NPV	Negative Predictive Value
NS	Statistically not Significant
OR	Odds Ratio
OSA	Obstructive Sleep Apnea
PPV	Positive Predictive Value
ROC	Area Under The Receiver Operating Curve
SDB	Sleep Disordered Breathing
SENS	Sensitivity
SPEC	Specificity

## Appendix A

**BAMSA**

**Questionnaire for screening sleep apnea**

(1) Is your **B**MI (body mass index) > 30 kgm^−2^?
  Yes   □ (1 point)   No   □ (0 points)
(2) **A**ge: Are you over 50 years old?
  Yes   □ (1 point)   No   □ (0 points)
(3) What is you gender?
  **M**ale   □ (1 point)   Female   □ (0 points)
(4) Do you **s**nore while sleeping (ask other people if you are not sure) at least one night per week?
  Yes   □ (1 point)   No   □ (0 points)
(5) Have you at least sometimes had breathing pauses (sleep **a**pnea) during sleep (or have other people noticed that you have pauses in respiration when you sleep)?
  Yes   □ (1 point)   No   □ (0 points)

If you had at least 3 points, your risk of having sleep apnea is elevated.
